# OLDER BLUES MUSICIANS

**DOI:** 10.1093/geroni/igz038.2892

**Published:** 2019-11-08

**Authors:** Michael Marcus

**Affiliations:** Consultants for Community Resources, Raleigh, N.Carolina, United States

## Abstract

Older Blues musicians have provided inspiration to generations of younger musicians as well as their own contemporaries.

